# Extraintestinal Manifestations of Celiac Disease: Early Detection for Better Long-Term Outcomes

**DOI:** 10.3390/nu10081015

**Published:** 2018-08-03

**Authors:** Pilvi Laurikka, Samuli Nurminen, Laura Kivelä, Kalle Kurppa

**Affiliations:** 1 Celiac Disease Research Center, Faculty of Medicine and Life Sciences, University of Tampere, 33014 Tampere, Finland; laurikka.pilvi.l@student.uta.fi; 2 Department of Internal Medicine, Hospital District of South Ostrobothnia, 60200 Seinäjoki, Finland; 3 Tampere Center for Child Health Research, Tampere University Hospital and University of Tampere, 33014 Tampere, Finland; nurminen.samuli.j@student.uta.fi (S.N.); laura.kivela@fimnet.fi (L.K.)

**Keywords:** celiac disease, extraintestinal, recognition, diagnosis, clinical presentation, gluten-free diet, prognosis

## Abstract

Population-based screening studies have shown celiac disease to be one of the most common chronic gastrointestinal diseases. Nevertheless, because of the diverse clinical presentation, the great majority of patients remain unrecognized. Particularly difficult to identify are the multifaceted extraintestinal symptoms that may appear at variable ages. Although the pathogenesis and long-term outcome of these manifestations are still poorly established, there is some evidence that unrecognized celiac disease predisposes to severe complications if not diagnosed and prevented with an early-initiated gluten-free diet. Therefore, it is of utmost importance that physicians of different disciplines learn to recognize celiac disease in individuals with non-gastrointestinal symptoms. In the future, more studies are needed to clarify the factors affecting development and prognosis of the extraintestinal manifestations.

## 1. Introduction

During the past few decades, we have come to recognize that celiac disease is among the most common gastrointestinal diseases, both in children and adults. The true prevalence of the disease in many Western countries is estimated to be as high as 1–3% and is increasing [1]. At the same time, modern sensitive and specific transglutaminase 2 (TG2) antibody based serological tests have made non-invasive case finding and screening of celiac disease considerably easier. In theory, the simplified diagnostics should have also increased the proportion of clinically detected cases, and this has indeed been the case in some, particularly Northern European, countries [2,3]. However, even in these areas, let alone globally, celiac disease remains seriously underdiagnosed. For example, it is estimated that in the United States, more than 90% of all affected patients are unrecognized [4]. It must also be emphasized that, instead of clinical case finding, a substantial proportion of patients in the aforesaid countries are found by at-risk group screening [5].

The evident difficulty in identifying celiac disease may be explained by in part by the heterogeneous and often vague clinical presentations. While the characteristic malabsorptive disease and gastrointestinal symptoms are relatively well known, the majority of celiac disease patients may in fact suffer from extraintestinal manifestations. These may affect almost every organ of the body, including the nervous system, liver, skin, and reproductive and musculoskeletal system [6,7,8,9]. Furthermore, some of these manifestations appear in early childhood, while the rest are not seen until adulthood, including in the elderly. Although evidence is limited, some extraintestinal presentations may lead to permanent complications if not recognized and treated early enough. Therefore, extraintestinal celiac disease needs to be recognized by physicians of various specialities, including gastroenterologists, internists, pediatricians, neurologists, dermatologists, gynecologists, and particularly general practitioners and family doctors.

In this review, we will provide an overview about current understanding of the pathogenesis and age-related clinical outcomes of the variable extraintestinal manifestations of celiac disease. In particular, we aim to improve the early recognition and diagnostic yield of this multifaceted condition, and subsequently reduce the risk of ill health and long-term complications.

## 2. Changes in Clinical Presentation

Celiac disease was long considered as a rare malabsorptive disease affecting mainly infants and toddlers. Classical symptoms included failure to thrive, chronic diarrhea, and abdominal distention. In the 1970s, the incidence of ‘typical’ celiac disease patients seemed to decrease and concurrently, older patients with milder symptoms were identified. When screening for celiac disease with sensitive and specific endomysium and transglutaminase antibodies from serum became possible in the 1980s and 1990s, respectively, the high prevalence and wide clinical presentation of celiac disease started to become evident [5,10].

Gastrointestinal related symptoms and signs such as anemia, impaired growth, decreased bone mineral density, and micronutrient deficiencies were recognized from early on to be associated with untreated celiac disease. Although these were initially thought to be present only in connection with malabsorption, it was later understood that they could also be a sole manifestation [11,12,13]. Dermatitis herpetiformis was among the first extra-intestinal symptoms of celiac disease recognized [14]. Later, other organ systems were found to be affected, when, for example, patients with earlier unexplained neurological and articular symptoms were diagnosed. In addition, as a result of the increased screening, the symptoms common in the general population, such as headache and tiredness, were often recognized in celiac disease patients. However, their true association to the disease and especially the response to the dietary treatment remains controversial [15]. Altogether, it is important to remember the unspecificity and complexity of the above-mentioned symptoms, as they may be present also in many other common disorders, such as irritable bowel syndrome (IBS), chronic fatigue syndrome, and migraine.

## 3. Variable Definitions for Symptoms and Findings 

Clinical features of celiac disease are often classified categorically as ‘typical’ and ‘atypical’. However, the definition for these terms is heterogeneous, which likely reflects the above-described historical background. Extra-intestinal manifestations are particularly difficult to classify, even though they may actually be even more common than the ‘typical’ symptoms. Consequently, a few years ago, a group of experts encouraged the alternate terminology of ‘classical’ and ‘non-classical’ celiac disease [16].

**Figure 1:**
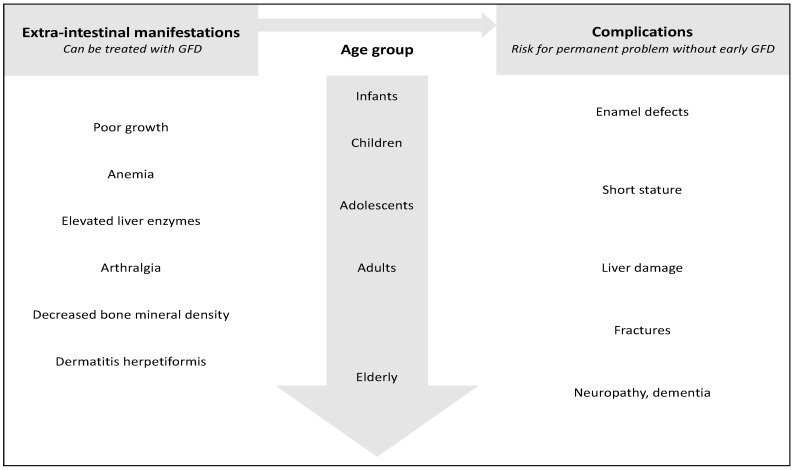
Extra-intestinal manifestations and complications of celiac disease classified based on their response to early initiated gluten-free diet (GFD) and typical age of development.

Another challenge in celiac disease is to discriminate the symptoms from the complications and from the independently associated diseases. Celiac disease symptoms should be reversible with the gluten free diet, whereas complications may be permanent and irreversible, especially if the initiation of treatment is delayed (Figure 1). Well-known associated conditions with an increased risk for celiac disease are for example type 1 diabetes, autoimmune thyroidal disease, and Down’s syndrome [17]. Whether the early diagnosis and treatment of celiac disease could prevent some of these conditions has been debated [18,19]. Again, these conditions should not be confused with totally unrelated disorders with often overlapping symptoms, such as IBS. 

## 4. Epidemiology and Pathogenesis of Extraintestinal Manifestations 

Although the existence of extraintestinal manifestations in celiac disease has been known for decades, their prevalence remains poorly established. This issue is complicated by the variable definitions and considerable age-related variations in the appearance of these symptoms (Table 1). For example, poor growth and delayed puberty are evidently exclusively pediatric presentations, whereas osteoporosis, infertility, and dermatitis herpetiformis are typical adulthood findings. Anemia, liver abnormalities, and joint problems are seen in both pediatric and adult patients (Table 1). Although celiac disease is known to be more common in females, the gender distribution of the extraintestinal symptoms is currently mostly obscure [20].

The pathogenesis of most extraintestinal manifestations is also obscure. There is some evidence that their presence is associated with an overall more severe clinical and histological presentation [20], but neither the appearance nor severity seems to be just a direct consequence of intestinal damage [21,22,23,24,25,26]. Another intriguing question is the balance between genetic and environmental factors, as even identical twins with celiac disease may have completely different phenotype [27]. The at least partially different pathogenic background of the variable presentations of celiac disease is further supported by the peculiar appearance of serological markers in some of these conditions [6,22,24]. The clinical features and plausible pathophysiological mechanisms of the best-characterized extraintestinal manifestations are further discussed below.

### 4.1. Poor Growth

Poor growth is likely one of the most common extraintestinal manifestations in pediatric celiac disease, although defining its true prevalence is complicated by its variable definitions (Table 1). We recently observed that impaired growth might be particularly common in children with a younger age at diagnosis and a more severe overall presentation of the disease [11]. For example, the malabsorption of nutrients and abnormalities in growth hormone-insulin-like growth factor axis and/or in thyroid function have been suggested as pathogenic mechanisms [26,38,39], but further studies are needed to confirm these findings.

### 4.2. Anemia

Anemia is a common extraintestinal manifestation of untreated celiac disease in all age groups (Table 1). Like poor growth, the presence of anemia seems to be associated with a more severe disease presentation [12,34]. Its prevalence could be expected to decrease over time as a result of an earlier diagnosis and the generally improved nutritional status of children. Regardless, celiac disease is still increasingly found in subjects with unexplained anemia [5]. With regards to pathogenesis, malabsorption/deficiencies of iron, vitamin B12, and folic acid may be implicated [40]. However, the etiology of anemia in celiac disease might be more complex, as it may be present in autoantibody positive individuals even before the development of enteropathy [41].

### 4.3. Neurologic Manifestations

Approximately one-fifth of celiac disease patients suffer from neurological manifestations (Table 1). The most common of these are gluten ataxia and peripheral neuropathy [42], which often present without accompanying gastrointestinal symptoms [43]. The pathogenesis of neurological manifestations is still somewhat obscure. In gluten ataxia, gluten-dependent transglutaminase 6 (TG6) autoantibodies targeted against the cerebellar cells may play a role and might be useful in the diagnostics of this condition [6,22]. However, TG6 antibodies have also been detected in children with celiac disease without association to neurological symptoms [23]. It remains to be proven whether this could predict the later adult onset of neurological symptoms.

### 4.4. Dental Enamel Defects

Disturbances in the amelogenesis of permanent teeth is a well-defined finding in celiac disease, but there are considerable variations in its reported prevalence (Table 1). Enamel defects have been particularly common in older studies together with severe infant celiac disease and lower general health [36]. It is possible that this manifestation is disappearing as, for example, is rickets related to celiac disease. However, for an accurate diagnosis the dental enamel should be evaluated by an expert dentist. Malnutrition, hypocalcemia, and immunologic disturbances have been suggested as causative factors of enamel defects [35], and the severity seems to be associated with the duration of gluten-exposure [44].

### 4.5. Liver Abnormalities

Prevalence of hypertransaminasemia has been reported in older studies up to 57%, while more recent studies report only 9–14% (Table 1), possibly again reflecting earlier diagnosis and milder presentation of celiac disease. Usually the hepatic injury is mild and easily reversible, but in rare occasions, celiac disease may lead even to liver failure [45]. The severity of hypertransaminasemia seems to be associated with the presence of malabsorption, high values of celiac autoantibodies, and advanced duodenal lesion [8,46], and the suggested mechanisms include altered gut permeability leading to an increased exposure to hepatotoxins in the portal circulation [47]. Interestingly, TG2 autoantibody deposits have been found in liver biopsies from affected patients and could contribute to the hepatocellular damage [21]. It is also important to keep in mind that celiac patients have overrepresentation of coexisting liver diseases such as autoimmune hepatitis, primary biliary cholangitis, and primary sclerosing cholangitis [48]. 

### 4.6. Joint Manifestations

Variable joint symptoms are quite often, although again inconsistently, reported in celiac disease (Table 1). The observed symptoms are usually described vaguely as arthralgia rather than objective synovitis [20,49]. However, subclinical synovial effusion and sacroiliitis have also been reported [50,51,52]. Differential diagnosis is challenging and includes, for example, growing pains in children and variable musculoskeletal complaints and arthrosis in adults. Once again, it should be remembered that celiac disease is associated with many autoimmune conditions, including rheumatologic diseases such as Sjögren’s syndrome [53], juvenile rheumatoid/idiopathic arthritis [54], and systemic lupus erythematosus [55]. At present, the pathogenesis of the joint symptoms in celiac disease remains entirely speculative.

### 4.7. Dermatitis Herpetiformis

Dermatitis herpetiformis is a dermatological manifestation of celiac disease that usually appears in adulthood (Table 1). The characteristic itching and blistering rash appears mostly on elbows, knees, and buttocks. In contrast to intestinal celiac disease, dermatitis herpetiformis is more common in males than females [7]. The diagnosis is based on skin biopsy showing characteristic IgA deposits in the papillary dermis next to the lesion [56]. Interestingly, besides TG2, dermatitis herpetiformis patients can also develop autoantibodies targeted against epidermal transglutaminase 3 [24]. Even though the characteristic rash is the primary manifestation, up to 72% of the patients also have enteropathy [57]. Interestingly, the incidence of dermatitis herpetiformis seems to be decreasing in contrast to other forms of celiac disease [7]. Furthermore, ‘classical’ celiac disease patients with poor dietary adherence can change their phenotype to dermatitis herpetiformis over time [58]. 

### 4.8. Bone Disease

Rickets is a classical finding in children with celiac disease, but it is nowadays rarely seen in developed countries. On the contrary, osteoporosis is common in adult celiac disease patients (Table 1), particularly in elderly patients and in postmenopausal women. The malabsorption of calcium and vitamin D may lead to secondary hyperparathyroidism and subsequently a high bone turnover and osteoporosis [59]. Increasing the circulating cytokines and an altered balance of bone turnover have also been suggested to play a role [60,61]. Neutralizing autoantibodies against osteoprotegerin have been detected in celiac disease patients [25], but their true role in the development of osteoporosis remains contradictory [62]. Decreased bone mineral density can also be seen in screen-detected patients [63], even before the development of enteropathy [64]. These observations emphasize the importance of early diagnosis of celiac disease to prevent advanced bone disease and subsequent fractures.

### 4.9. Problems in Reproductive and Endocrine Systems 

Delayed puberty can be observed in children with untreated celiac disease [15,32], and various problems in reproductive health, such as infertility, recurrent miscarriages, and intrauterine growth restriction, have been reported in adult female patients [9,15]. The pathogenesis of delayed puberty in celiac disease is unknown, although it is a common finding also in many other chronic diseases during adolescence. However, there are several hypotheses for the pathogenesis of reproductive system’s maladies. Untreated celiac disease may lead to deficiencies of micronutrients such as zinc, selenium, and folic acid which are important during pregnancy and fetal development, anemia may also have a role [9]. Celiac disease may affect other hormonal systems, for example, TG2 autoantibodies are able to bind to TG2 in thyroid tissue and the celiac autoantibody titers have been shown to correlate with the antithyroperoxidase antibody titers [65]. However, this should not be confused with the overrepresentation of celiac disease among children with autoimmune thyroid disease due to a shared genetic background [66]. 

### 4.10. Other Extraintestinal Manifestations

Besides the above-mentioned, several other extraintestinal manifestations have been suggested to be associated with celiac disease. Aphtous ulcers, headache, and fatigue are frequently reported but unspecific clinical findings [15,31,35]. Several neuropsychiatric conditions including learning disorders, developmental delay, and attention deficit hyperactivity disorder have also been associated with celiac disease [31], as well as are psychiatric disorders including depression, anxiety, and even schizophrenia [15,67]. In addition, there are reports about possible associations between celiac disease and uveitis, eczema, psoriasis, asthma, and various cardiovascular symptoms [68,69,70,71]. However, because of the common and non-specific nature of these conditions, a true pathogenic relationship to celiac disease requires further evidence [15,72]. 

## 5. Effects of Dietary Treatment to the Extraintestinal Manifestations

In general, a gluten-free diet is an effective treatment for celiac disease. However, the dietary response might be faster and more complete in children than in adults [73,74]. Extraintestinal manifestations also have a good prognosis in children if appropriately treated with a gluten-free diet (Table 2) [8,73,75], but in adults and in a subgroup of children, the dietary response might be incomplete [12]. In such cases, the possibility of coexisting conditions should be remembered. Dapsone medication is often needed as an additional therapy in dermatitis herpetiformis for some time [75].

A gluten-free diet has also been shown to be beneficial for more advanced extraintestinal manifestations, such as a low bone mineral density, liver failure, and infertility [9,13,46,76,77,78], but here, the timing of the diagnosis is crucial (Table 2). Early developing complications such as dental enamel defects should actually be treated even before their appearance [44]. In children with poor growth, a significant catch-up growth is usually achieved after the beginning of dietary treatment and thus reduced adult height is uncommon [78,79]. However, growth problems should likely be treated at latest in puberty, in order to avoid suboptimal height development [80,81,82]. With regards to bone health, patients diagnosed in childhood seem to have no increased risk for osteoporotic fractures [83], but again, in order to achieve optimal bone accrual, an early diagnosis is beneficial [84].

Non-adherence is the most common reason for an unsatisfactory treatment response [15,73]. Nevertheless, some extraintestinal manifestations may not improve entirely despite an apparently strict diet, which may reflect their more complex etiology [15,72,73], but it is also possible that at least a part of them are in fact related to disorders other than celiac disease. Accordingly, Jericho et al. found that almost 30% of the children with a failure of catch-up growth on a gluten-free diet to have some co-existing condition [15]. Therefore, additional reasons for poor response should be sought in cases with proven adherence. True pediatric refractory celiac disease is almost non-existent and one should be very cautious before making such a diagnosis [86]. 

## 6. Importance of Early Diagnosis

As discussed, it seems that most of the advanced extraintestinal manifestations might be preventable by the early diagnosis of celiac disease. One way to increase the diagnostic yield would be screening of at-risk groups. Counterweighting this approach is the burden of a gluten-free diet, which is challenging to maintain, socially restrictive, and expensive. This may lead to reduced quality of life particularly in patients with negligible symptoms. There is, however, some evidence that even apparently asymptomatic adults may already have advanced histological disease and benefit from the diet [63,87]. Furthermore, a long diagnostic delay may increase the risk of poor clinical response [73,88,89]. This issue is less well defined in screen-detected children, but there is data showing that they may also experience unrecognized symptoms, including extraintestinal manifestations [90,91], and benefit from an early diagnosis [91,92,93,94]. 

An unsolved question is that a part of screened individuals may have a so-called potential celiac disease with positive celiac autoantibodies but normal villi, and thus do not fulfil the current diagnostic criteria [95]. This might be an early stage of developing celiac disease, but at present, more evidence about the natural history of this condition is warranted [96]. 

Population-based mass screening would be a very effective approach to finding unrecognized celiac patients, but is currently not recommended because of the lack of scientific evidence [95,97,98]. Major open questions concerning such a wide-scale screening are the above-mentioned challenges in the diagnostics of patients with early or potential celiac disease, and the balance of the benefits and harms of the treatment for asymptomatic patients. Furthermore, the cost-effectiveness of screening is particularly important in these circumstances, but, unfortunately, this issue is currently very scarcely studied. 

All in all, the current recommendations about screening for celiac disease have been varying between countries and between children and adults [95,98,99]. However, most organizations recommend a targeted at-risk group screening, this usually including first-degree relatives of celiac disease patients and those suffering from type 1 diabetes [95,99]. Meanwhile, before further evidence about screening is provided, active case finding is especially important. By carrying this out efficiently, at least part a of the patients with extraintestinal manifestations could be diagnosed before appearance of permanent complications. 

## 7. Conclusions 

In recent years, we have witnessed an upsurge of interest towards different gluten-related conditions, demonstrated, for example, by the rapid increase of the popularity of self-adopted gluten-free lifestyle. This notwithstanding, the clinical prevalence of officially diagnosed celiac disease, is all but satisfactory. This is worrisome, as the condition is proven to be associated with an increased risk of long-term complications that could very likely be prevented by timely diagnosis and initiation of a gluten-free diet. 

As wide-scale serological screening is currently not recommended, we should aim to improve the physicians’ and other allied health professionals’ knowledge of the diverse presentations of celiac disease. For pediatricians, this especially means the identification of the manifestations that can present already at early childhood and may lead to permanent problems if undetected. Then again, many extraintestinal symptoms may appear later in life and should thus be known by physicians of relevant adult subspecialties. It is important to acknowledge the critical role of primary healthcare in these circumstances, as only effective first-line case finding can lessen the ill-health caused by untreated celiac disease at the population level. Therefore, we should educate particularly the primary care providers, in order to understand the value of the early detection of this multifaceted condition.

In the future, we need more information on the prevalence, age of appearance, dietary response, and long-term prognosis of the variable extraintestinal features of celiac disease. Moreover, at present, the details of the pathogenesis and reasons for the substantial phenotype heterogeneity in celiac disease remain mostly obscure. This information could, besides improving the diagnostics of celiac disease, also provide novel insight of the development of autoimmune diseases in general. 

## Figures and Tables

**Table 1:** Prevalence of the best-characterized extraintestinal manifestations of untreated celiac disease in children and adults.

	Children	Adults	
	%	%	References
Poor growth ^1^	11–70	-	[20,28,29]
Short stature ^1^	4–33	3	[15,30,31,32,33]
Anemia	12–40	23–48	[12,15,20,28,30,32,33,34]
Neurological symptoms	4–52	24	[15,20,28,29,31]
Enamel defects	0–15	1–83	[20,29,32,33,35,36]
Liver abnormalities	1–57	2–5	[8,15,32]
Joint manifestations	1–10	2–9	[15,20,29,30,32,33]
Dermatitis herpetiformis	2–3	10–20	[20,30,32,37]
Osteoporosis	0	4–23	[15,32]
Infertility	-	5	[9,15]

^1^ Definitions of poor growth and short stature have varied between the studies.

**Table 2:** Response of extraintestinal manifestations in celiac disease to a gluten-free diet (GFD).

Manifestation	Response	Comments	References
Anemia	Yes	Sometimes slow or incomplete response	[12,15]
Dermatitis herpetiformis	Yes	Dietary response may be slow and require additional Dapsone medication	[15,75]
Transaminasemia	Usually	Often mild and reversible; in rare cases may lead to liver failure	[8,45]
Poor growth	Variable	May lead to reduced adulthood height if not treated before puberty	[15,82]
Neurological symptoms	Variable	In children, usually good response, but in adults, possibly irreversible	[6,43]
Decreased bone mineral density	Variable	Initiation of GFD before school age may be needed for optimal bone accrual	[13,76,84]
Joint problems	Variable	Coexisting musculoskeletal disease should be excluded if poor response	[15]
Enamel defects	Infrequently	Early appearance and irreversible in permanent teeth	[44]
Infertility	Unclear	Conflicting results	[9,77,85]
